# The characterization and phylogenetic analysis of complete chloroplast genome in *Morella cerifera* (Myricaceae)

**DOI:** 10.1080/23802359.2020.1845997

**Published:** 2021-01-11

**Authors:** Yuanlin Guan, Pengkai Wang, Hongli Qie, Yinghong Huang, Wensheng Yu

**Affiliations:** a Taihu Extension Center for Evergreen Fruit of Jiangsu Province, Suzhou, China; b Suzhou Polytechnic Institute of Agriculture, Suzhou, China; c College of Horticulture, Vegetable Genetics and Breeding Laboratory, Anhui Agricultural University, Hefei, China

**Keywords:** *Morella cerifera*, chloroplast genome, Myricaceae, phylogeny

## Abstract

The first complete chloroplast genome of *Morella cerifera* was obtained by illumina platform sequencing technology in this study. The size of genome is 158,943 base pairs, consist of a pair of IRs 26,043 bp in length, the LSC region of 88,167 bp and SSC region of 18,690 bp. The genome has 112 unique genes, among which 79 protein-coding genes, 29 tRNAs, and 4 rRNAs. Phylogenetic analysis revealed that *M. cerifera* clustered with *M. rubra* within Myricaceae.

## Introduction

*Morella cerifera* (L.) Small is the species of the genus *Morella* within the family of Myricaceae (Chen et al. 2004). *M. cerifera* is an evergreen dioecious plant, symbiotic with nitrogen-fixing bacteria (Frankia SP) and can grow on relatively barren sandy and alkaline soils (Zhizhen 2003; Karumuna et al. 2019). *M. cerifera* is one of the pioneers in afforestation improvement in saline and alkaline lands. It has the medicinal value of analgesia and treatment of digestive problems (Zhang et al. 2016; Makule et al. 2018). Chloroplast, plant-specific organelles with special genetic material, named chloroplast genome (Meng et al. 2018), is the site of plant photosynthesis, which can provide energy for plant synthesis of starch, amino acids and other substances (Neuhaus and Emes 2000). The number and sequence of genes in chloroplast genome are relatively conservative, and there is good collinearity among various plant groups (Huang et al. 2014; Lim et al. 2018; Wang et al. 2019). Chloroplast genome, due to its size variations, gene and intron losses, nucleotide substitutions, independent encoding specific proteins, non-recombinant and uniparental inheritance, usually has been used as ideal research model to study plant evolution and comparative genome (Liu et al. 2017). However, chloroplast genome of *M. cerifera* has never been reported. In this study, the chloroplast genome of *M. cerifera* was sequenced and constructed the phylogenetic analysis with other chloroplast genomes.

Plant materials were taken from Suzhou, China, located at 120.40E, 31.05 N. Voucher specimens (numbered as *Morella cerifera*-WF01) were collected from the Germplasm bank and were deposited at the Herbarium of Suzhou Polytechnic Institute of Agriculture (Herbarium Code: MCEM). Chloroplast DNA (cpDNA) was extracted from fresh leaves by the CTAB method (Doyle 1987), and the cpDNA was preserved after extraction (in Suzhou Polytechnic Institute of Agriculture). Genomic library was prepared with an insert size of 250 bp and the whole genome of *M. cerifera* chloroplast was sequenced using illumina platform sequencing technology (illumina NovaSeq 6000). About 5.7 G of raw reads were obtained and were filtered by the program Trimmomatic v.0.33 (Bolger et al. 2014). The chloroplast genome was assembled by using the filtered reads with the program Velvet 1.2.10 (Zerbino and Birney 2008). Gene was annotated online by *GeSeq-Annotation of Organellar Genomes* (https://chlorobox.mpimp-golm.mpg.de/geseq.html) (Tillich et al. 2017) and the annotated genome sequence was submitted to NCBI GenBank (Accession number MT872488). The analysis results showed that the chloroplast genome length of *M. cerifera* is 158,943 bp, comprising a pair of IRs 26,043 bp, separating the LSC region of 88,167 bp and SSC region of 18,690 bp. The nucleotide content was 36.17%(GC) and 63.83% (AT), while the IR region has a GC content of 42.58%, significantly higher than that of the LSC and SSC regions, which are 33.87% and 29.17%, respectively.

**Figure 1.**
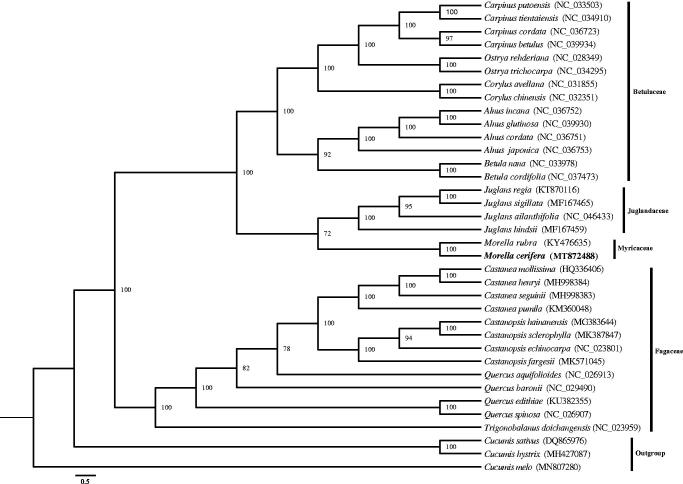
The phylogenetic relationships of *M. cerifera* based on complete chloroplast genomes. Numbers next to the branches are bootstrap support values. *Morella cerifera* is marked by bold and the accession number is listed alongside the species name.

There were 112 unique genes annotated in the whole chloroplast genome of *M. cerifera*, including 79 protein-coding genes, 4 rRNA genes and 29 tRNA genes. Five protein-coding genes (*rps*7, *ndhB*, *ycf*2, *rpl*23, *rpl*2), seven tRNA genes (*trnN-GUU*, *trnR-ACG*, *trnA-UGC*, *trnI-GAU*, *trnV-GAC*, *trnL-CAA*, *trnM-CAU*), and four rRNA genes were duplicated within the IRs. In addition, there were 16 genes containing introns, including 15 genes containing one intron and one gene containing two introns (*clpP*). Thirty-five chloroplast genomes were downloaded from NCBI, of which 32 fagale chloroplast genomes and 3 cucurbitale genomes were taken as outgroups to assess the phylogenetic position of *M. cerifera*. Phylogenetic tree was established by using the IQ-TREE v1.6.5 with the Maximum Likelihood (ML) method (Nguyen et al., 2015). The tree showed a relationship that *M. cerifera* clustered with *Morella rubra* in a unique clade in family Myricaceae (Figure 1). This study provides the phylogenetic status of *M. cerifera* with chloroplast genomic resources.

## Data Availability

The data that support the findings of this study are openly available in GenBank at https://www.ncbi.nlm.nih.gov/genbank/, reference number MT872488. The raw sequencing reads used in this study have already been deposited in a public repository (SRA) at https://www.ncbi.nlm.nih.gov/bioproject/PRJNA660127.
